# CAR-T Therapy, the End of a Chapter or the Beginning of a New One?

**DOI:** 10.3390/cancers13040853

**Published:** 2021-02-18

**Authors:** Yasser Mostafa Kamel

**Affiliations:** 1 School of Cancer & Pharmaceutical Sciences, Faculty of Life & Sciences & Medicine, King’s College, London SE1 9NH, UK; yasser@asyspc.com; 2 ASYS Pharmaceutical Consultants-APC Inc 2, Bedford, NS B4A 4L2, Canada

**Keywords:** chimeric antigen receptor-t, CAR-T therapy, haematologic malignancies, solid tumours, cytokine release syndrome

## Abstract

**Simple Summary:**

CAR-T therapy is a breakthrough treatment in our fight against cancer. It was recently approved for the treatment of advanced diffuse large B-cell lymphoma and acute lymphoblastic leukaemia after the failure of previous multiple therapies. The positive results achieved in the registration studies for those patients were remarkable. Unfortunately, this was not the end of this chapter. Disease relapses occur in the range of 30–60% of patients treated with CAR-T therapy. Cytokine release syndrome represents a major side effect for treatment with CAR-T therapy. Notwithstanding, the high positive results triggered the start of a huge research activity of CAR-T therapy in other haematologic malignancies such as acute myelogenous leukaemia, Hodgkin’s disease, chronic lymphocytic leukaemia, and multiple myeloma. The research is also trying to overcome the hurdles stated above. These activities represent a new chapter in the management of haematologic malignancies with CAR-T therapy.

**Abstract:**

Chimeric antigen receptor-T (CAR-T) therapy targeting CD19 has revolutionised the treatment of advanced acute lymphoblastic leukaemia (ALL) and diffuse large B-cell lymphoma (DLBCL). The ability to specifically target the cancer cells has shown high positive results as reported in the registration studies. The success of CAR-T therapy in the first two indications led to the initiation of a large number of studies testing CAR-T therapy in different haematologic tumours such as acute myelogenous leukaemia (AML), Hodgkin’s disease (HD), chronic lymphocytic leukaemia (CLL), multiple myeloma (MM), as well as different solid tumours. Unfortunately, relapses occurred in patients treated with CAR-T therapy, calling for the development of effective subsequent therapies. Likewise, this novel mechanism of action was also accompanied by a different toxicity profile, such as cytokine release syndrome (CRS). Patients’ access to the treatment is still limited by its cost. Notwithstanding, this did not prohibit further development of this new therapy to treat other malignancies. This research activity of CAR-T therapy moves it from being used as an end-stage treatment for ALL and DLBCL to a new therapeutic option for a wide range of patients with different haematologic and solid tumours.

## 1. Introduction

In 2017, the Food and Drug Administration (FDA) granted the first approvals of two CAR-T therapies for the treatment of adult patients with relapsed or refractory diffuse large B-cell lymphoma (DLBCL) after two or more lines of systemic therapies, including DLBCL not otherwise specified, primary mediastinal large B-cell lymphoma, high-grade B-cell lymphoma, and DLBCL arising from follicular lymphoma, and for patients up to 25 years of age with B-cell precursor acute lymphoblastic leukaemia (ALL) that is refractory or in second or later relapse [1,2].

The registration studies enrolled patients who were previously treated and considered of poor prognosis, with some having had prior stem cell transplantation. Yet, the results that were obtained from the studies (albeit single-arm studies) were quite encouraging and resulted in high responses in those difficult-to-treat patient populations [3]. The genetically engineered T cells, which are injected into the patient’s body, result in engraftment and rapid proliferation. Subsequently, each CAR-T cell attacks many cancer cells. This unique mechanism of action, with the involvement of the patient’s own immune system, resulted in a very high response rate despite the late disease stages and poor prognoses [4,5]. Furthermore, CAR-T therapy was used to bridge other therapeutic strategies with further improvement of responses. With the high response rates and durable responses with long-term follow-up [6], it was hoped that the responses would be for long durations with the possibility of a cure for these patients. Unfortunately, the relapse rate was around 30–60% for patients treated with CAR-T therapy [7]. With limited treatment options for patients who relapse after CAR-T therapy, it is of paramount importance to understand the mechanism of resistance in those patients in order to develop a subsequent effective therapy.

## 2. Mechanism of Relapse to CAR-T Therapy

Patients who relapsed following CAR-T therapy fall into two main groups of relapse: CD19-positive cells or CD19-negative cells [6]. The CD19-positive cells relapse occurs when the response to CAR-T therapy is not sufficient, with minimal effect for CAR-T cells and only transient B-cell aplasia. With flow cytometry, CD19 can be detected on the surface of the cells. Different animal studies attempted to identify the cause of this weak response to CAR-T therapy. Some of the factors that were proposed are the quality of CAR-T cells synthesised from children and young adults compared to those from older adults. For younger patients, the quality of the CAR-T cells was better, which was reflected in the tendency to receive better responses (longer event-free survival times) in the younger age groups versus older age groups. Studies have shown that the starting T-cell phenotype of CAR-T cell manufacturing is crucial to patients’ prognosis. A study conducted by Gardner et al. enrolled 43 children and young adults [7]. They looked for factors that contributed to a maintained response among those who responded, achieved, and maintained a minimal residual disease (MRD)-negative state beyond 63 days, versus the non-responders who did not achieve that [7,8]. The analysis of apheresis obtained from both groups showed that in non-responders, the percentage of CD8+ T cells, which expressed lymphocyte-activated gene-3 (LAG-3) and PD-1, had significantly increased, whereas in the responders group, the number of CD4+ CAR cells and CD8+ CAR cells was significantly higher at the time of peak implantation. They also found that the number of CD8+ T cells expressing tumour necrosis factor-α (TNF-α) was much less in the non-responder group compared to the responder group. This led to the assumption that the increase in LAG-3-expressing cells (LAG-3+ T cells) and decrease in the cytokines after stimulation leads to the production of ineffective CAR-T cells, or ones with low potency resulting in a CD19-positive relapse. This is in comparison to the responders who have less LAG-3-expressing cells and an increase in cytokines after stimulation, thus having a better response [9]. The presence of LAG-3+ T cells is a biomarker for T-cell exhaustion. T-cell exhaustion refers to a state where the T cells produced are dysfunctional with the decrease in the effectors and increase in the expression of the inhibitory receptors. This phenomenon is induced by chronic stimulation in patients with cancer. Chronic stimulation leads to the production of T cells that are usually deficient in their intrinsic activity. Accordingly, CAR-T cells derived from patients with cancer will be defective in function, which can lead to poor prognosis and relapse. The mechanism of CAR-T cell exhaustion is still not clearly understood. It was suggested that the CARs on the CAR-T cells could form a cluster that is formed independently of the antigen stimulation, which leads to the development of tonic CAR-CD3ζ signalling that can cause CAR-T cell exhaustion. Additionally, the endogenous T-cell receptor signal of CAR-T cells caused by a specific antigen stimulation can result in T-cell exhaustion [6,9].

The CD19-negative relapses represent up to 20% of the relapsed CAR-T cell treated patients. With CD19-negative relapse, CD19 is not detected on the surface of the cells. This occurs due to mutation in exons 1–13 of the CD19 gene [10]. Exons 1–4 carry the codes for the extracellular domains, and exons 5–13 carry those of the transmembrane domain. Studies have shown that patients who show CD19-negative relapse have developed mutations in exons 2–5 in the form of one insertion or deletion [11]. The percentage of CD19-negative cells can be measured by flow cytometry, which is also able to assess the frequency of allelic mutations and the percentage of cells where there is a homozygous loss and biallelic mutations, which are major reasons for the loss of the targeted epitope in the membrane of CD19, with the subsequent escape from the effect of anti-CD19 CAR-T. Called antigen escape, this leads to the development of resistance to CD19-directed CAR-T therapies.

There are other mechanisms that were described as possible causes for the occurrence of CD19 gene mutation and antigen escape, such as alternative splicing with the subsequent development of CD19-negative resistance [12]. These resistance mechanisms are not unique to CAR-T therapy. They have been observed in other tumour types, e.g., breast cancer tissues show splicing of exon 16 of HER2 leading to resistance to trastuzumab. Likewise, melanoma tissues show splicing of BRAF (V600E), which results in dimerisation and consequently resistance to vemurafenib. Splicing of mRNA (e.g., in SF3B1, SRSF2) also occurred in haematological tumours such as myelodysplastic syndrome (MDS) and chronic lymphocytic leukaemia (CLL) resulting in resistance to treatment [13].

## 3. CRS: A Limit to the Full Therapeutic Potential of CAR-T Therapy?

Both approved CAR-T cells cause the condition called cytokine release syndrome (CRS), which includes a set of life-threatening or even fatal reactions to the infusion of CAR-T therapy. Generally, CAR-T therapy should not be administered to patients who have active infections or inflammatory disorders.

**Figure 1:**
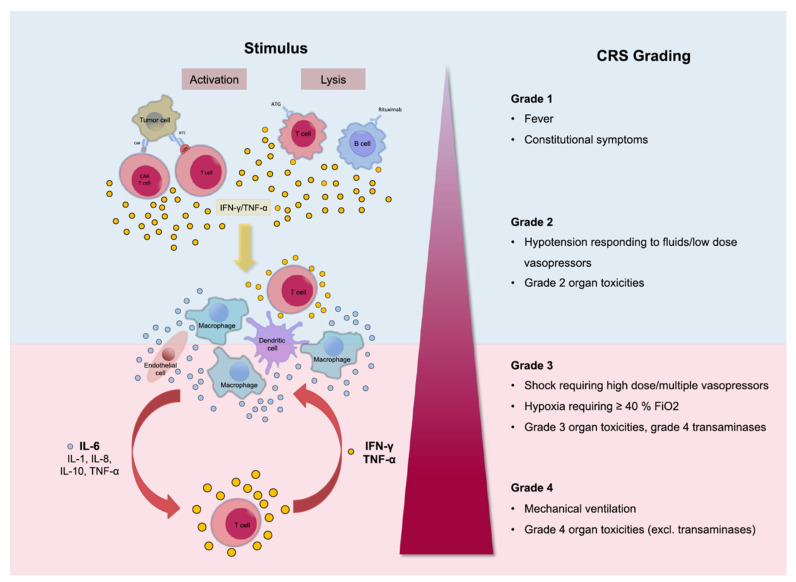
Pathophysiology of cytokine release syndrome (CRS) with the activation of different cytokines. The clinical picture can differ from mild, only representing with fever (Grade 1), to life-threatening conditions (Grade 4). Reprinted from Alexander Shimabukuro-Vornhagen et al. J Immunother Cancer 2018;6:56 with permission from the Journal for ImmunoTherapy of Cancer [16].

Treatment with CAR-T cells causes rapid activation of T cells and the release of high levels of cytokines. The main cytokines involved in CRS are IL-6, IL-10, and interferon (IFN)-Υ. IFN-Υ, when secreted, causes the release of other cytokines such as IL-6, IL-10, and TNF-α. Its release is also accompanied by fever, chills, headache, malaise, and other symptoms occurring with CRS. IL-6 also is considered a key cytokine in the pathophysiology of CRS since it is highly elevated in cases suffering from CRS. It is also responsible for the occurrence of many of the severe symptoms such as vascular leakage and disseminated intravascular coagulation (DIC). The occurrence of life-threatening CRS mandates immediate treatment with tocilizumab or tocilizumab and corticosteroids. [1,2,14]. Tocilizumab, an anti-IL-6 receptor antagonist, is the standard for CRS management, although the optimal timing of administration is still unclear [14,15] (Figure 1). Management is also depending on the severity of the symptoms. The commonly used scheme for the assessment of the severity of CRS is the one developed by the National Cancer Institute (NCI).

CRS can affect different body systems and organs. Other medical specialties such as neurology, nephrology, cardiology in addition to the treating haematologist are required for the effective management of patients with severe CRS. Intensive care facilities should also be available when needed. The early and appropriate management of severe cases of CRS generally can lead to a positive outcome [15].

CRS can affect different organs, and the symptoms can range from mild to severe and debilitating [16]. Respiratory symptoms that are common with CRS can range from cough and hypoxia to acute respiratory distress syndrome, which can be seen on lung images as opacities affecting large areas of the lung and can be bilateral [16,17,18].

Cardiac and circulatory manifestations can be in the form of hypotension and can progress to capillary leak syndrome with subsequent peripheral and pulmonary edemas and signs of cardiac failure [19].

**Figure 2:**
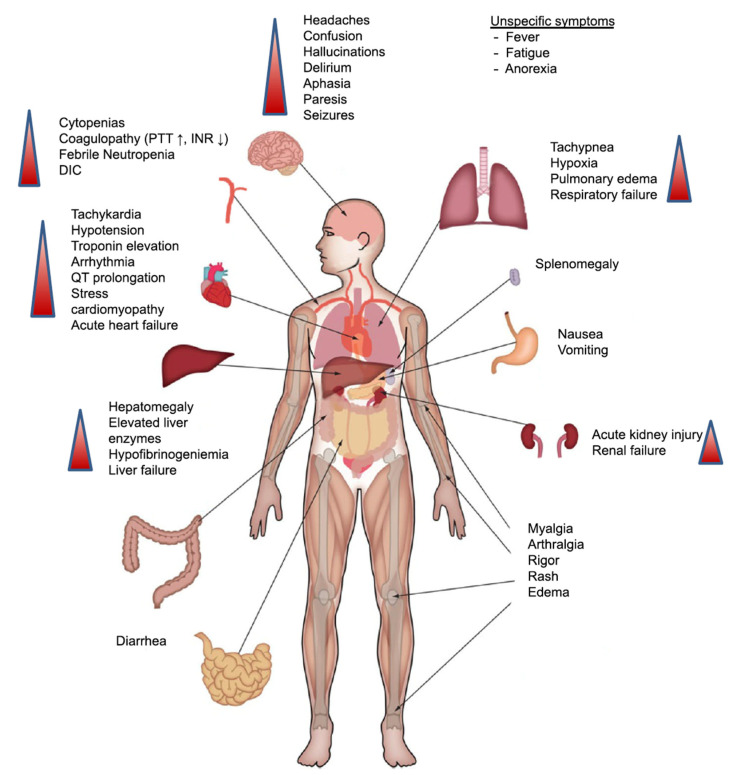
Clinical presentation of CRS. Symptoms can range from fever to the affection of multiple organs. Abbreviations: DIC: disseminated intravascular coagulation; INR: international normalised ratio; PTT: partial thromboplastin time. Reprinted from Alexander Shimabukuro-Vornhagen et al. J Immunother Cancer 2018;6:56 with permission from the Journal for ImmunoTherapy of Cancer [16].

Neurotoxicity is also a serious type of toxicity that can occur following treatment with CAR-T therapy [20]. It can be in the form of mild confusion symptoms and headaches to a severe form of hemiparesis, cranial nerve palsies, and even seizures [21]. As a result of the relatively common incidence of neurotoxicity and the seriousness of it, the term CAR-T cell-related encephalopathy syndrome (CRES) is now used [22]. Despite the common incidence of CRES (being the second most common toxicity associated with CAR-T therapy) and the severity of the symptoms, neither the exact pathophysiology [23] nor the best way to treat it is clear; thus, the management of it may need to be carried out in specialised care units [24,25] (Figure 2).

Investigators have attempted to decrease the CRS signs/symptoms of CAR-T therapy while maintaining its positive therapeutic effect. Frey et al. conducted a study on 35 adults with relapsed/refractory (R/R) ALL who received CAR-T therapy [26]. Patients were enrolled in 1 of 3 dosing cohorts. The first cohort was a low-dose cohort where nine patients received a low dose of therapy either as a single dose or fractionated dose. In the second cohort, six patients received CAR-T therapy as a high single dose, and the third cohort had 20 patients enrolled and received high dose therapy either as a single dose or fractionated dose. Any fractionated dose was given on 3 consecutive days with 10%, 30%, and 60% given on days 1, 2, and 3, respectively. This schedule allowed for the assessment of CRS occurrence, taking necessary measures if they occur, and trying to avoid fatal complications.

The nine patients in the low single or fractionated dose had a complete remission (CR) rate of 33% with manageable toxicity. In the six patients who received the high single dose, three had fatal CRS complications, and the other three achieved CR. The results in the third cohort, where patients received a fractionated high CAR-T dose, were impressive with a CR rate of 90%, an event-free survival rate (EFS) of 49.5% (95% CI: 21–73%), and a 2-year overall survival (OS) rate of 73% (95% CI: 46–88%). Superior results were achieved for the patients who were dosed with a high fractionated dose of CAR-T cells, whereas the toxicity was manageable with only one patient out of the 20 patients having CRS grade 4 and no grade 5 CRS. The fractionation of CAR-T over 3 days allowed for early detection of clinical symptoms of CRS and withholding of consequent doses. Accordingly, in the third cohort, 7 out of the 20 patients received all three doses, nine patients received only one dose, and four patients received two doses. Only two patients in this cohort did not achieve CRS, and both only received two doses. The authors concluded that CAR-T cell fractionation allowed for early dose modification and consequently optimising the safety of patients with R/R ALL who received CAR-T therapy without compromising the efficacy of treatment [26].

Gardner et al. tested the early intervention of tocilizumab plus or minus corticosteroids with the first occurrence of mild CRS symptoms in a study of 43 patients. The first 23 patients received tocilizumab with or without corticosteroids if there was an occurrence of any grade 4 toxicity not meeting the study’s defined dose-limiting toxicity (DLT) and lasting >48 h, or there was a DLT lasting >48 h, and in either situation, the CRS was not effectively managed by medication. Corticosteroids were also given to any non-haematologic toxicity >grade 3 that was considered a result of CAR-T infusion, lasting for >48 h, and not controlled despite active medical intervention. The subsequent 20 patients received concomitant tocilizumab and corticosteroids every 6–12 h for persistent mild CRS symptoms according to the requirement of a protocol modification. The investigators reported that despite the patients being treated with tocilizumab and steroids, there was no increase in the incidence of infection. Likewise, the efficacy was not affected by the early treatment with tocilizumab and steroids or the expansion and engraftment of T cells. They also reported a decrease in severe CRS in the 20 patients who had early intervention and concomitant tocilizumab and corticosteroids, versus the first 23 patients who had their CRS managed as above (15% vs. 20%) [27].

In conclusion, the therapeutic effect of CAR-T therapy is well established and has been proven in a wealth of studies; however, the fact that there is a high percentage of patients suffering from relapse and CRS presents serious complications for treatment with CAR-T therapy. It represents huge challenges for the treating physicians and the patients. Ongoing research activity is trying to address the mechanism of relapse and the best way to administer CAR-T therapy in order to reduce the incidence of CRS [28,29].

## 4. CAR-T Therapy in Other Haematologic Malignancies: Is There a Role?

The unique mechanism of CAR-T therapy in addition to the high positive results mentioned above also triggered wide development programmes assessing this novel therapy in different haematologic malignancies as well as solid tumours.

### 4.1. CAR-T Therapy in AML

In 2020, the American Cancer Society estimated AML to represent 1% of all cancers. It is mainly a disease of the older population, being uncommon before the age of 45, with the average age at first presentation of 58 years. Its incidence is slightly more common among men than women. It is the most common leukaemia in adults and the second most common leukaemia in children [30,31].

Currently, with the standard 7 + 3 regimen (consisting of cytarabine for 7 days and anthracycline for 3 days), a CR rate of up to 80% has been achieved in young adults and up to 60% in older adults who are 60 years of age and above. This is followed by post-remission induction, which differs according to different factors, e.g., patient’s age, general condition, molecular prognostic stratification, etc. [32,33]. However, patients who do not achieve remission from the first-line regimens and those who relapse pose a serious problem for the haematologists with the continuous need for the development of effective therapies [34].

In ALL, the presence of a specific target such as CD19 supported the scientific concept behind the development of a specific targeted CAR-T therapy. However, the lack of a specific cell target for AML makes it difficult to treat this disease with targeted immunotherapy [35,36]. Currently, different researchers are attempting to identify an antigen or a group of antigens that are predominantly expressed on the myeloblast and not the normal tissues. This concept of specifically targeting surface antigens on myeloblasts is important to be able to target AML disease while avoiding the exposure of normal tissues to unnecessary immunotherapy, which could result in toxicity [37].

The concept of using targeted immunotherapy for patients with AML was successfully applied by treating CD33-positive AML patients (adults and children over 2 years of age) with chemotherapy and the CD33-targeted antibody-drug conjugate, gemtuzumab ozogamicin [38,39]. The positive results of this combination led to the approval of this combination in adults and children over 2 years of age who suffer from CD33-positive AML [40,41].

**Figure 3:**
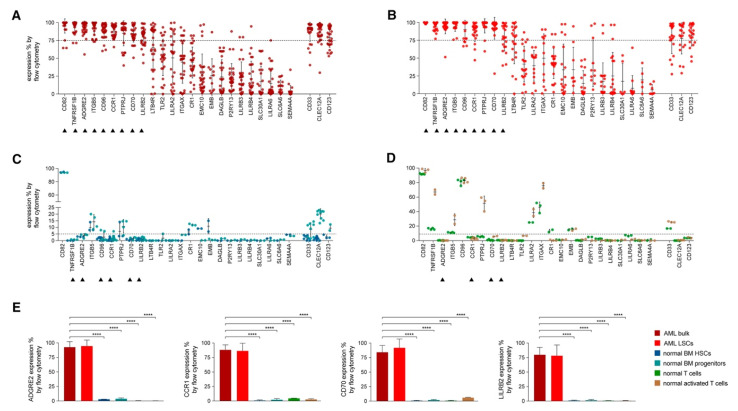
Flow Cytometric Analyses in Primary AML Samples and Normal Haematopoietic Cells. (**A**–**D**) Expression (% positive) of the 24 candidate antigens and the three CAR targets in current clinical investigation (most right three) in bulk AML population (**A**), in leukaemic CD34^+^CD38^−^ cells (**B**), in normal BM CD34^+^CD38^−^ CD45RA^−^ CD90^+^ HSCs (blue), CD34^+^ CD38^+^ progenitor cells (light blue) (**C**), or in freshly purified (green) or activated (brown) normal CD3^+^ peripheral blood T cells (**D**). Data are represented as mean ± SD. (**E**) Summary of expression levels (mean ± SEM) of four top targets in indicated cell populations. **** *p* < 0.0001 (Student’s *t*-test). Reprinted from Perna F, Berman SH, Soni RK, et al. Integrating proteomics and transcriptomics for systematic combinatorial chimeric antigen receptor therapy of AML. Cancer Cell 2017; 32: 506–519.e505 with permission from Elsevier [42].

In an attempt to identify the ideal candidates for CAR-T therapy in AML, Perna et al. conducted an extensive analysis of large datasets of transcriptomics and proteomics from malignant and normal tissues [42] (Figure 3). The researchers performed surface-specific proteomic studies in a diverse panel of AML cell lines (THP1, Mono-mac, Kasumi, Molm13, OCI/AML3, and TF-1). The team performed a mass spectrometric analysis, which led to the identification of 4942 proteins. They enriched their protein sets by adding the findings from other similar studies conducted on other AML cell lines (e.g., NB4, HL60, THP1, PLB985, CD32, CD33, CE 96, CD99, and K562). The researchers used data from the Human Protein Atlas, the Human Proteome Map, and the Proteomics Database to identify the protein expression information in different normal tissues/organs such as liver, gallbladder, pancreas, stomach, gut, rectum, testis, epididymis, prostate, breast, etc. [43,44,45]. Consequently, they looked for CAR-T therapy targets that are overexpressed in AML versus normal tissue, and they were able to identify 24 molecules that fulfil this criterion (Figure 3A,B). Using flow cytometry to analyse the expression of the 24 candidates in AML specimens, they managed to detect nine targets that are detected in >75% of the cells (expression range: 78–99%, mean 82%): CD82, TNFRSF1B (also known as CD120b), ADGRE2 (also known as EMR2 or CD312), ITGB5, CCR1 (also known as CD191), CD96, PTPRJ (also known as CD148), CD70, and LILRB2 (also known as CD85d). Six of the nine targets were expressed in <5% in normal tissues (with ADGRE2, CCR1, CD70, and LILRB2 expressed in <5% in freshly purified and activated T cells from healthy donors), whereas the other three were expressed in the normal tissues at a slightly higher level with the maximum expression of 20% (Figure 3C,D). Still, all were much less expressed than their corresponding expression in the primary AML cells. (Figure 3E) [42].

Many pre-clinical studies were also conducted to assess the different possible targets for CAR-T therapy in AML populations, which led to the start of the Phase I clinical studies. These studies tested a wide range of ages, from as young as 6 months to up to 90 years of age. The number of patients treated in these studies was small. Cummins et al. treated six patients with CAR-T therapy. Two patients achieved complete remission (CR), three patients achieved partial remission (PR), and one patient had progressive diseases (PD) [46]. Richie et al. also treated four patients with CAR-T, with all of them initially achieving PR or stable disease (SD), then they relapsed [47]. Of note, both Cummins et al. and Richie et al. used autologous T-cell sources. Numerous studies are ongoing, with data still to be presented [48,49].

### 4.2. CAR-T Therapy in CLL

In order to assess if CAR-T therapy can target CLL cells, a group of researchers from the NCI in Bethesda managed to construct two CARs. They subsequently chose the one that has shown the best anti-tumour activity in vitro for further testing in CLL clinical trials [50].

One of the earliest observations of clinical activity in CLL was reported by Porter et al. in 2011 [51]. A patient who had R/R CLL was infused with a low dose (approximately 1.5 × 10^5^ cells per kilogram) of autologous CAR-T cells. A real-time polymerase chain reaction detected DNA encoding anti-CD19 CAR-T after 1 day of the infusion of the cells. The infused CAR-T cells expanded to a level more than 1000 times the initial engraftment. The patient achieved CR. The toxicity profile was as expected, with the only grade 3/4 adverse event related to CAR-T therapy being lymphopenia as well as hypogammaglobulinemia, which was a chronic effect. The patient also suffered from tumour lysis syndrome. Leukaemia, as well as normal B cells-expression CD19, disappeared from the patient’s blood and bone marrow cells. A high level of CAR-T cells was maintained in the patient’s blood and bone marrow for 6 months, and the patient’s remission was ongoing at 10 months post-engraftment [52].

Following that, CAR-T therapy was investigated in different clinical trials, with more than 130 patients being tested in the trials. Most of these patients were heavily pre-treated, some relapsed post haematopoietic cell transplant [53,54]. Others were cases treated as they progressed to Richter syndrome. Despite the heterogeneity of the patient population and the adverse prognostic factors, a CR rate in the range of 20–30% of the patients with estimated progression-free survival (PFS) at 18 months of 25% was achieved. These studies have shown the potential activity of CAR-T therapy in the CLL patient population [55,56].

Ibrutinib, which is a breakthrough treatment for patients with CLL, was found to improve response in clinical trials. In one of the trials, treatment with CAR-T therapy following ibrutinib in three patients resulted in responses in all three patients, with one achieving CR [57]. Two other studies combining ibrutinib and CAR-T therapy have shown very promising response rates of 80% in two series of 19 patients with an MRD eradication in the bone marrow of around 90% among responders. The safety profile was not different from that of other patients with other indications when treated with CAR-T therapy, with the CRS being the main concern [58,59]. Other studies are ongoing to explore the full potential of CAR-T therapy in patients with CLL [60].

### 4.3. CAR-T Therapy in Hodgkin Lymphoma (HL)

Data from two studies testing CAR-T therapy targeting the CD30 antigen in patients with HL were recently presented in the Transplantation and Cellular Therapy Meetings of American Society for Blood and Marrow Transplantation (ASBMT) and Center for International Blood & Marrow Transplant Research (CIBMTR) and highlight the potential role of anti-CD30 CAR-T cells for this disease [61,62]. In one of the trials, the investigators treated the patients with a lymphodepletion chemotherapy that included cyclophosphamide and fludarabine (FC) before CAR-T cell infusion. With 14 patients enrolled in this Phase I study, the median age was 30 years (range: 17–69 years). Patients were CD30 positive HL and heavily pre-treated with a median of five prior regimens. Most patients previously received a checkpoint inhibitor and the monoclonal antibody brentuximab targeting CD30. Patients received a single infusion of one of three dose levels: 2 × 10^7^ cells/m^2^, 1 × 10^8^ cells/m^2^, or 2 × 10^8^ cells/m^2^. The investigators found the expansion and persistence of the CAR-T cells to be dose-dependent. Of the 14 patients treated, important safety findings were seen in four patients who developed grade 1 CRS, and some patients developed maculopapular rashes that disappeared without any treatment. Other adverse events were in the form of alopecia, gastrointestinal toxicities, and transient cytopenias. At 6 weeks, 12 patients were evaluable for response, seven patients achieved CR, and one patient achieved PR. One of the CR patients showed the response by 6 weeks and maintained the remission for more than a year [61].

In another Phase I/II study, patients received one of two dose levels in a standard 3 + 3 design. The doses tested in Phase I were 1 × 10^8^ cells/m^2^ or 2 × 10^8^ cells/m^2^, and the Phase II part of the study tested the selected Phase I dose in more patients [62]. In both Phases of the study, a total of 29 patients were enrolled with a median age of 35 years (range: 23–69 years). All patients had a refractory disease and were heavily pre-treated with a median of eight previous regimens. There were 28 evaluable patients, of which 26 (8 in Phase I and 18 in Phase II) received CAR-T cell infusion (24 patients had classic HL, and the other two had T-cell lymphomas). In the eight patients enrolled in the Phase I part of the study, three patients received the lower dose of CAR-T cells (1 × 10^8^ cells/m^2^) and all progressed. Of the other five patients who received the high dose (2 × 10^8^ cells/m^2^), three patients had CR, one patient had SD, and one patient had PD. As there were no dose-limiting toxicities, the 2 × 10^8^ cells/m^2^ was selected for the Phase II part of the study. Bendamustine (B) single agent was given for lymphodepletion in Phase I of the study. However, as response and CAR-T cell expansion were suboptimal, the investigators decided to add fludarabine (F), i.e., bendamustine and fludarabine combination (BF).

Out of the 18 patients treated in Phase II, four developed CRS (three grade 1 that resolved spontaneously, and one was grade 2 that was managed by tocilizumab). There were also nine patients who had mild rashes after the infusion. Patients also had cytopenias in the form of grade 3 or high neutropenia in 3 patients (12%), thrombocytopenia and lymphopenia each occurring in four patients (15%). Of the 18 patients who received CAR-T cell infusion, 14 patients achieved CR (78%), of which two had a response longer than 1 year. Another two patients (11%) achieved PR; one patient had SD (5%). Only one patient had PD (6%). Of note, the CR rate was higher in the patients who had the combination of BF (78%) versus the patients who received B monotherapy (37%), with survival also being longer in those who had BF than those who had F alone (median of 389 days vs. 55 days). After a median follow-up of 108 days (range not reported), progression-free survival was 164 days for 19 evaluable patients who had active disease at the time of lymphodepletion. Patients who received the higher dose of CAR-T cells with the combination lymphodepletion regimen appeared to have a longer survival than those treated with a lower dose and single-agent bendamustine (median = 389 days vs. 55 days; *p* = 0.0004) [62].

These data suggest the potential role of CAR-T therapy targeting CD30 HL. However, further studies with larger numbers of patients and longer follow-up periods are needed to identify the role of anti-CD30 CAR-T cell infusion in HL.

### 4.4. CAR-T Therapy in MM

Multiple myeloma (MM) is a plasma cell cancer, and despite the recent advances in treatment and the use of immunomodulatory, proteosome inhibitors, and other novel therapies, there is still no cure for it [63,64]. Current available therapies improve disease outcomes, but patients will ultimately relapse. Each relapse negatively affects survival chances for the patients [65,66].

Superior results are seen with CAR-T therapy in non-Hodgkin lymphoma and ALL encouraged the testing of CAR-T cell therapy in the disease of MM. B-cell maturation antigen (BCMA) is a protein and a tumour necrosis factor that is expressed by normal and malignant plasma cells including MM cells [67,68]. A specific CAR-T cell therapy (bb2121) was produced by the transduction of autologous T cells with a lentiviral vector that encodes a second-generation CAR-T that incorporates an anti-BCMA single-chain fragment, a CD137 (4–1BB) costimulatory motif, and a CD3-zeta signalling domain [69,70].

CAR-T therapy has shown the potential to benefit patients with MM. In a pre-clinical Phase I study conducted by Raje et al., bb2121 (a CAR-T agent that targets BCMA) showed promising activity in patients with R/R MM [71]. The study consisted of two phases: dose escalation and dose expansion. In the dose escalation phase, a single infusion of CAR-T cells was tested in groups of patients in a dose-escalating manner. The doses that were tested were 50 × 10^6^, 150 × 10^6^, 450 × 10^6^, and 800 × 10^6^. In the dose expansion phase, only two doses were tested: 150 × 10^6^ and 450 × 10^6^. The study enrolled heavily pre-treated patients who received at least three previous lines of therapy (the median was 7 and the range was 3–14). With the exception of one patient, all patients previously failed an autologous stem cell transplant. All patients previously received and relapsed or were refractory to an immunomodulatory agent and a proteasome inhibitor. Some patients also had extramedullary disease (27%), and 45% of the patients had a high-risk cytogenetic profile with the presence of t(4;14), t(14;16), or del(17p). Safety was the primary endpoint of the study, and the safety results of 33 evaluable patients (out of 36 enrolled in the study) were reported. The main toxicity was haematologic toxicity, with events of grade 3 or higher reported for 85% of the patients in the form of neutropenia. Other grade 3 or higher toxicities were reported in the form of leukopenia, anaemia, and thrombocytopenia in 58%, 45%, and 45% of the patients, respectively. The non-haematologic toxicities were in the form of CRS, which occurred in 25 patients (76%). Most of the patients (23 patients, 70%) had a maximum of grade 2 CRS, and only two patients (6%) had grade 3 CRS. Neurologic toxicities occurred in 14 patients (42%), with only one patient suffering from a reversible grade 4 event and all the remaining patients having only grade 1 or 2. A CR was achieved in 15 patients (45%), one patient had PR (3%), and a median of PFS of 11.8 months was achieved. Of note, six patients out of the 15 patients ultimately relapsed. It is important to note that some responders had MRD negativity. The investigators concluded that BCMA-directed cellular immunotherapy for patients with R/R MM had toxicities similar to what was reported in other CAR-T cell therapies used for other indications. The therapy also resulted in promising responses in the form of CR/PR with doses of 150 × 10^6^ in this heavily pre-treated patient population of R/R MM [71].

In another first-in-human study (FIH), the anti-B-cell maturation antigen BiTE molecule was assessed in patients with R/R MM [72]. Patients who relapsed after at least two prior lines of therapies and no extramedullary disease were treated in the study. Patients received up to 10 cycles of treatment, given as an infusion every 4 weeks. The length of the cycle was 6 weeks. MRD was assessed using flow cytometry, and MRD negativity was defined as the presence of <1 cell/10^4^ bone marrow cells. A total of 42 patients were enrolled in the study. The median age of the patients was 65 years. Patients had a median duration of MM of 5.2 years. The median number of cycles received by the patients was 1 (range: 1–10 cycles), while the responders received a median of 7 cycles. The doses received by the patients were in the range of 0.2–800 µg/d. Reasons for treatment discontinuation were disease progression in 25 patients (60%), adverse events in seven patients (17%), and death in four patients (10%). Likewise, three patients (7%) withdrew from treatment as they received the maximum allowable cycles in the study (10 cycles), one patient (2%) withdrew consent for continuation of treatment, and two patients (5%) were still on treatment at the time of the presentation of these data. Safety results have shown that the 800 µg/d was not tolerated, with two out of the three patients enrolled having a DLT, with one having a grade 3 CRS and the other one suffering from neurotoxicity (polyneuropathy) that started as grade 2 and progressed to grade 3. Both events were later resolved. For the patients who received the 400 µg/d dose, one patient had grade 1 polyneuropathy that progressed to grade 3 but ultimately resolved by week 12. CRS was seen in 16 out of the 42 patients (38%); however, it was treated with glucocorticoids, antihistamines, antipyretics, and analgesics, with one patient receiving tocilizumab for grade 2 CRS. Additionally, there were five patients who had increases in liver enzymes (ALT and/or AST > 3× times the upper limit of normal). Among them, four patients received the 400 µg/d. However, one of the four patients who had increased liver enzymes had increased enzymes at baseline. At the 400 µg/d dose, which was received in 10 patients, seven achieved a response rate of 70%. Among the seven responders, there were five patients who achieved MRD negative CRS. For the remaining two patients, one had a PR, and one had a very good PR. All the responses occurred in the first cycle, and some responses lasted >1 year. The investigators concluded that these results are promising and warrant further investigation [72].

The CARTITUDE-1 was a Phase Ib/II trial that enrolled adult patients who were heavily pre-treated with three or more prior lines of therapy or were double-refractory to an immunomodulatory drug and a proteasome inhibitor. Patients received a single low dose of ciltacabtagene autoleucel (cilta-cel), an autologous CAR-T therapy. Cilta-cel is a bioengineered T-cell receptor construct with a CD3ζ signalling domain, a 4-1BB costimulatory domain, and two BCMA binding domains. The Phase I study objectives were safety and defining a dose to carry forward to the Phase II part of the study. Patients underwent apheresis and cell collection to produce the CAR-T cells. The patients had lymphodepletion with 3 days of fludarabine 30 mg/m^2^ plus cyclophosphamide 300 mg/m^2^. The dose received of cilta-cel was a single dose of 0.75 × 10^6^ cells/kg (range: 0.5–1.0 × 10^6^ cells/kg). At the time of the cut-off level of 1 September 2020, 113 patients were enrolled, with 97 receiving CAR-T (Phase Ib 29 patients and Phase II 68 patients). The median age of the patients was 61 years (range: 43–78 years), and the median number of prior lines of therapies was 6 (range: 3–18). Haematologic adverse events were the most common, with neutropenia, anaemia, and thrombocytopenia occurring in 96%, 81%, and 79% of the patients, respectively. Most haematologic adverse events resolved quickly without complications. Non-haematologic adverse events were CRS (95% of patients) and neurotoxicity (21% of patients). Most CRS events were grade 1/2 (95%). All CRS events resolved within 14 days of their onset in all but one patient. Only 10% of the patients who suffered from neurotoxicity had their events as grade ≥3. The number of deaths was 14, five of which were due to PD, three due to adverse events unrelated to the CAR-T therapy, and six due to adverse events related to the therapy. The efficacy results have shown that 67% of the patients achieved stringent CR, 26% achieved a very good PR, and 4% had PR. There were 57 patients who were evaluable for the assessment of MRD, 93% of whom became MRD negative. PFS at 12 months was 77%. The investigators recommended the testing of this CAR-T therapy in a larger clinical trial [73].

The efficacy and safety of Ide-cel (bb2121) in patients with relapsed and refractory multiple myeloma and in subjects with high-risk multiple myeloma (KarMMA Phase II study) also reported the results of Ide-cel, another CAR-T therapy targeting BCMA. Based on the Phase I promising activity, the Phase II study enrolled 140 heavily pre-treated patients with MM. The median age of the patients was 61 years. For lymphodepletion, the patients received cyclophosphamide 300 mg/m^2^ and fludarabine 30 mg/m^2^ for 3 days. This was followed by the infusion of CAR-T cells in the range of 150–450 × 10^6^. Of the 128 heavily pre-treated patients (median of prior 6 regimens) who received Ide-cel, and with a median follow-up period of 11.3 months, the overall response rate ORR (the primary endpoint of the study) was 73%, and the PFS was 8.6 months. The most common adverse events of any grade were cytopenias and CRS occurring in 97% and 84% of the patients, respectively. CRS was mainly grade 1/2, with only 5% having grade 3, one patient having grade 4, and one patient dying of CRS at the 300 × 10^6^ dose of CAR-T. The response was durable, with 36% having CAR-T cells detected at 12 months. The investigators concluded that Ide-cel has demonstrated deep and durable responses in this heavily pre-treated patient population with an acceptable safety profile [74].

## 5. What Is Next?

The previously discussed studies in different haematologic malignancies represent a huge opportunity for patients who are suffering from those diseases. The fact that most of these studies are early phase studies with promising results and that there are planned Phase III studies represents great research opportunities for the scientific community and a chance for patients to be treated with a highly effective CAR-T therapy. These opportunities come with some challenges; the CRS is one of them. Some ongoing studies try to minimise this side effect while maintaining the superior outcome seen with CAR-T therapy [26]. Cost still represents a challenge to treatment with CAR-T therapy. Treatment with CAR-T cells is still not affordable for many patients in the approved indications. With the potential of more indications to come, the cost of treatment, either for the patients or any health care system, should be thoroughly evaluated, which also includes the cost of hospitalisation during treatment with CAR-T therapy and possible costs for the management of side effects [75].

## 6. Conclusions

Treatment with CAR-T therapy has started a new concept for the treatment of patients with advanced ALL and DLBCL. The search for subsequent therapy for those who failed treatment with CAR-T therapy, together with the huge ongoing and planned studies in different haematologic malignancies, opens a new era of research and treatment with gene/targeted therapy. Although CAR-T therapy is currently approved for advanced/heavily pre-treated patient populations, the research activities will potentially be conducted in earlier disease settings. All the ongoing and planned activities lead to one conclusion: this is not the end of the chapter; it is the beginning of a new chapter or even chapters in our fight against different haematologic malignancies.

## Data Availability

No new data were created or analyzed in this study. Data sharing is not applicable to this article.
